# Dissecting the effect of heat stress on durum wheat under field conditions

**DOI:** 10.3389/fpls.2024.1393349

**Published:** 2024-06-28

**Authors:** Eder Licieri Groli, Elisabetta Frascaroli, Marco Maccaferri, Karim Ammar, Roberto Tuberosa

**Affiliations:** ^1^ Department of Agricultural and Food Sciences, DISTAL, University of Bologna, Bologna, Italy; ^2^ International Maize and Wheat Improvement Center, CIMMYT, El Batán, Mexico

**Keywords:** durum wheat, field condition, heat-stress, modeling, yield components

## Abstract

**Introduction:**

Heat stress negatively affects wheat production in several ways, mainly by reducing growth rate, photosynthetic capacity and reducing spike fertility. Modeling stress response means analyzing simultaneous relationships among traits affecting the whole plant response and determinants of grain yield. The aim of this study was to dissect the diverse impacts of heat stress on key yield traits and to identify the most promising sources of alleles for heat tolerance.

**Methods:**

We evaluated a diverse durum wheat panel of 183 cultivars and breeding lines from worldwide, for their response to long-term heat stress under field conditions (HS) with respect to non stress conditions (NS), considering phenological traits, grain yield (GY) and its components as a function of the timing of heat stress and climatic covariates. We investigated the relationships among plant and environmental variables by means of a structural equation model (SEM) and Genetic SEM (GSEM).

**Results:**

Over two years of experiments at CENEB, CIMMYT, the effects of HS were particularly pronounced for the normalized difference vegetation index, NDVI (-51.3%), kernel weight per spike, KWS (-40.5%), grain filling period, GFP (-38.7%), and GY (-56.6%). Average temperatures around anthesis were negatively correlated with GY, thousand kernel weight TKW and test weight TWT, but also with spike density, a trait determined before heading/anthesis. Under HS, the correlation between the three major determinants of GY, i.e., fertile spike density, spike fertility and kernel size, were of noticeable magnitude. NDVI measured at medium milk-soft dough stage under HS was correlated with both spike fertility and grain weight while under NS it was less predictive of grain weight but still highly correlated with spike fertility. GSEM modeling suggested that the causal model of performance under HS directly involves genetic effects on GY, NDVI, KWS and HD.

**Discussion:**

We identified consistently suitable sources of genetic resistance to heat stress to be used in different durum wheat pre-breeding programs. Among those, Desert Durums and CIMMYT’80 germplasm showed the highest degree of adaptation and capacity to yield under high temperatures and can be considered as a valuable source of alleles for adaptation to breed new HS resilient cultivars.

## 1 Introduction

Wheat (*Triticum* spp.) is one of the most important staple foods for the human diet worldwide. In 2022, wheat has been cultivated in 219.2 million ha worldwide with a total production of over 808.4 million tons (FAO, 2024, https://www.fao.org/faostat/en). This makes it the third most important crop in terms of global production. Wheat is cultivated across a wide range of latitudes, from 67° North to 45° South including highly diverse environmental conditions (FAO, 2021). About 10% of wheat production is attributed to durum wheat (*Triticum durum* Desf.), a species of strategic importance in Mediterranean countries. The lower genome content of tetraploid wheat as compared to hexaploid wheat makes durum wheat a simplified proxy to better understand the complex, highly quantitative response of polyploid wheat to environmental factors at a physiological and molecular level (Adamski et al., 2020).

Wheat plays a crucial role in the human diet (Shewry and Hey, 2015), but different environmental conditions, including high temperatures, frequently cause significant yield losses in this crop (Pequeno et al., 2021). The current situation is predicted to become worse, especially due to climate change effects and the resulting increase in temperatures. The Intergovernmental Panel on Climate Change (IPCC) predicts that the increase in temperature will range from 2.7 to 4.8°C by 2100 (IPCC (IPCC, 2023). Semenov and Shewry (2011) predicted that heat stress (HS) will have a stronger negative impact on wheat production in Europe than drought stress. Indeed, it has been reported that, on average, each 1°C of further temperature increase will reduce grain yield in wheat by 6% (Graziani et al., 2014; Asseng et al., 2015; Martre et al, 2017; Zhao et al., 2017).

Heat stress (HS) negatively affects wheat in several ways. It imposes a reduction in photosynthetic capacity (Feng et al., 2014; Posch et al., 2019), an alteration in the plant water relations (Hasanuzzaman et al., 2013) and metabolic activities (Farooq et al., 2011), increases the production of reactive oxygen species (Sharma et al., 2019; Chen and Yang, 2020), and reduces spike fertility and grain filling when occurring at flowering or during the grain filling period (Akter and Islam, 2017; Balla et al., 2019; El Hassouni et al., 2019; Fabian et al., 2019; Balla et al., 2021). At the vegetative stage, the primary effect of HS on wheat is a reduction of seed germination, potentially leading to a poor stand establishment (Hossain et al., 2013; Akter and Islam, 2017). When occurring at tillering, HS negatively affects the survival/growth of fertile tillers, significantly compromising the yield potential of the crop (Poudel et al, 2021; Poudel et al., 2022). According to Porter and Gawith (1999), HS can reduce the number of fertile tillers by 15.38%. Additionally, HS impairs meristem development and reduces plant growth due to leaf senescence and abscission (Balla et al., 2019), and reduction of photosynthesis (Posch et al., 2019) leading to lower biomass production as well as reduced grain yield.

Although HS has a significant impact on plants during vegetative stages (seedling growth, tillering and stem elongation), it is during the reproductive stages (booting, flowering, and grain filling) that has a more pronounced effect on plant development, fertility, and crop performance (Akter and Islam, 2017; Hussain et al., 2018; Telfer et al., 2018; Balla et al., 2019; Fabian et al., 2019; Sharma et al., 2019; Balla et al., 2021). According to Porter and Gawith (1999), the negative effect of HS depends on the timing, duration, and magnitude of the stress imposed on the plants. The optimal temperatures for plant growth and function vary depending on the developmental stage of the plant. The optimal temperature for wheat growth is around 21°C during anthesis and grain filling. In an extensive review, Porter and Gawith (1999) reported that wheat can withstand up to 31°C at anthesis and 35.4°C during grain filling, when even a short period of stress might seriously damage grain yield and yield components. Additionally, HS at anthesis reduces pollen fertility and/or its viability and growth leading to poor fertilization as well as abnormal ovary development, hence reducing seed setting and the number of kernels per spike (Djanaguiraman and Prasad, 2014; Fabian et al., 2019; Ullah et al., 2022) hence grain yield directly. After anthesis (‘terminal stress’), HS reduces thousand kernel weight and volumetric weight (Rehman et al., 2021).

Depending on the timing and duration, HS can directly affect plant development, hence phenology and more specifically tillering, time of heading and flowering, particularly under conditions of early HS and relatively high night and day temperatures during early development (Mamrutha et al., 2020). On the opposite, variation for heading date caused by genetic determinants other than heat stress (e.g., photoperiod response, vernalization response), indirectly affects HS response and should be considered as a co-variate, rather than a directly causing the physiological response (Tuberosa, 2012). As pointed out by van Eeuwijk et al. (2019) traits related to phenology such as flowering time can make it difficult to evaluate and dissect the genetic control of other traits that are indirectly influenced by phenology itself. Moreover, breeders are interested in defining the causal relationship among the traits involved in stress tolerance, to define which traits to consider for predicting breeding values. This knowledge can be also be deployed to reduce the number of traits to consider for GY prediction (Abdalla et al, 2021; Powell et al., 2021).

Structural Equation Models (SEM) (Wright, 1921) can be applied to study relationships among phenotypes in multivariate systems and can produce an interpretation of relationships among traits different from that obtained with standard multiple trait models, where all relationships are represented by linear associations among random variables. Unlike in multiple trait models, in SEM a given trait can be treated as a predictor of another one, providing a functional (causal) link between both (Rosa et al., 2011). SEM has been described and used in quantitative genetics models (Gianola and Sorensen, 2004), pre-selecting the causal relationships based on prior knowledge. More recently, Valente et al. (2013) proposed searching for recursive causal structures in the context of mixed models for the genetic analysis of multiple traits, showing that it may be possible to infer phenotypic networks and causal effects even without QTL or marker information (Rosa et al., 2011). In SEM a primary trait can be modeled in terms of its component traits, as in factorial regression and crop growth models, but they are also suitable for modeling other biological components of traits (van Eeuwijk et al., 2019) which can be considered in designing an ideotype, helping breeders to define a selection strategy. Network models can be extended to multiple environments since variation in genetic correlations between traits across environmental conditions is an important cause of G×E and SEM could make such changes visible in a biologically meaningful way (Topner et al., 2017; Momen et al., 2019; van Eeuwijk et al., 2019; Kruijer et al., 2020; He et al., 2021).

This study was designed to evaluate the response to crop cycle-long heat stress under field conditions with respect to non stressed optimal conditions in a diverse durum wheat panel from different countries. The aim of this study was to dissect the diverse impacts of heat stress on key yield traits and to identify the most promising sources of alleles for heat tolerance. We considered phenological traits, grain yield and its components as a function of the extent of heat stress and weather covariates. We investigated the use of different SEMs to identify a network of traits that could help in the identification of heat tolerant genotypes. We took into account the genetic population structure and origin of the analyzed germplasm, drawing on a collection representing genetic diversity from around the world, to identify those to be considered as suitable sources of genetic tolerance to heat stress for use in durum wheat breeding programs.

## 2 Materials and methods

### 2.1 Genetic materials

The plant material used in this study consisted of a durum wheat diversity panel (namely UNIBO-Durum Panel) of 183 accessions (cultivars and elite breeding lines) from Mexico (CIMMYT), USA and Mediterranean countries (Italy, Morocco, Spain, Syria, Tunisia), which were selected from a larger panel (336 accessions) based on their pedigree and heading date to minimize variation due to phenology while maximizing variation in origins. Accordingly, accessions with high identity-by-descent value based on pedigree and molecular markers data (Maccaferri et al., 2007b; Maccaferri et al, 2007a) and/or with differences higher than 7 days in heading date in Mediterranean countries (Maccaferri et al., 2011) were excluded to reduce possible bias caused by phenology. Additional information about UNIBO-Durum Panel is reported in (Maccaferri et al., 2005; Maccaferri et al., 2007a; Maccaferri et al., 2007b);. Additionally, five elite cultivars used as parental lines in different Recombinant Inbreed Lines (RILs) populations at CIMMYT and the University of Bologna were added to the experiment in order to evaluate their response to HS (**Supplementary Table 1**) (Condorelli et al., 2018).

### 2.2 Environment characterization

The field experiment was carried out at the Campo Experimental Norman E. Borlaug (CENEB), CIMMYT’s experimental station, near Ciudad Obregon (Sonora) located in northwest Mexico (27° 33’ N; 109° 09’ W; 38 masl) (Honsdorf et al., 2020). The weather at the CENEB station is characterized by an arid climate with highly variable rainfall (Verhulst et al., 2011). The annual mean temperature is approximately 23.5°C ranging from 16.0°C in January to 31.0°C in July (Honsdorf et al., 2020). Based on the World Reference Base (Verhulst et al., 2009), classified the soil at CENEB location as a Hyposolic Vertisol (Calcaric, Chromic), with low soil organic matter (SOM< 12 g kg^-1^ of soil) as well as slight alkalinity (pH 8) which is well within the non-toxic range for wheat. According to the world Mega-Environments (MEs) classification system developed by CIMMYT, the CENEB location includes ME1 (temperate, completely irrigated optimal conditions) when wheat is sown at optimal time (November 15-December 15) and ME5 (high heat exposure with non-water limiting conditions) when wheat is planted late (February 15-March 1) for heat tolerance evaluation (Mondal et al., 2013). Meteorological data from the 2017/18 and 2018/19 were collected at a meteorological station approximately 2 km from the experimental area.

### 2.3 Experimental design and field evaluation

The UNIBO durum panel was evaluated in two crop seasons (2017/18, 2018/19 – referred to as 2018 and 2019 hereafter, respectively) as well as two different environmental conditions, namely (i) control (non-stressed NS) with optimal sowing date, last week of November/first week of December, and (ii) heat-stressed (HS) with late sowing date, last week of February/first week of March to expose plants to higher than the normal temperature during their entire cycle.

The experiment was carried out in a complete randomized block design with two replicates, arranged in a rectangular grid with 10 rows by 56 columns. Each experimental unit (plots) consisted of two rows of 2.1 meters accounting for a plot area of 1.68 m^2^. Plot management, including fertilizer regimes, weed, pest, and disease control followed CIMMYT agronomic practices to optimize growing conditions regardless of testing environment and maintain plots free of weeds and diseases or pests. To avoid any confounding effects due to drought stress, plants received at least four auxiliary irrigations per season using a furrow irrigation system.

The following traits were measured and are described in details in **Supplementary Table 2**: days to heading (HD), days to maturity (DTM), grain filling period (GFP), plant height (PH), number of spikes per linear meter (SPM), kernel number per spike (KNS), kernel weight per spike (KWS), number of spikelets per spike (SKT), grain yield (GY), thousand kernel weight (TKW), test weight (TWT), kernel length (KLE) and kernel width (KWI). Additionally, as a proxy for total biomass, normalized difference vegetation index (NDVI) was estimated on different dates during the whole cycle for both experiments (NS, HS) and for both crop seasons. NDVI was estimated with individual measurements during the vegetative and grain filling growth stages as reported in **Supplementary Table 3**. NDVI was measured by canopy reflectance using the GreenSeeker RT100 equipment (Optical Sensor Unit, NTech Industries, Inc.). After performing all individual measurements, for each experiment we chose the single most significant NDVI measurement identified by ANOVA to represent the trait, which corresponded to the reading taken at Zadoks stage 75–83, corresponding to medium milk-soft dough stage (**Supplementary Table 3**) (Zadoks et al, 1974). Furthermore, the four most significant NDVI measurements were used to derive an index (IT_NDVI), calculating the area under NDVI progress curve by adapting equation (Equation 1) reported in (Simko and Piepho, 2012).


(1) $$\text{IT}\_\text{NDVI}=\sum_{i=1}^{n-1}\left((\frac{NDVI_{i}+\;NDVI_{i+1}}{2})*\left(GDD_{i+1}-GDD_{i}\right)\right)$$

Where: $NDVI_{i}$ is NDVI at *i* ^th^ measurement; $GDD_{i}$ is accumulated growing degree days at the *i* ^th^ measurement and *n* is the total number of measurements. GDD refers to the number of heat units, degree days in °C, required for a crop to progress from stage 1 to stage X. In this study we used the method proposed by Davidson and Campbell (1983) (Equation 2).


(2) $$GDD=\sum \;\left(\left(\frac{Tmax+Tmin}{2}\right)-Tb\right)$$

Where: GDD is growing degree days in °C; Tmax is maximum daily temperature; Tmin is the minimum daily temperature in °C; Tb is base temperature, for wheat is 0°C). The chosen NDVI measurements, either for the NDVI or for the IT_NDVI, were almost at the same GDD accumulation level (**Supplementary Table 3**).

### 2.4 Weather parameters

To monitor the environmental conditions specific of each genotype based on its specific phenological stage, four additional temperature parameters were calculated independently for each single plot within each experimental condition as described by (Telfer et al., 2018). According to Dreccer et al. (2008), the anthesis period is defined as the period from 300 GDD before to 100 GDD post-anthesis time. Similarly, the grain filling period is defined as the period from 100 GDD to 600 GDD post-anthesis time. We did not collect data for anthesis time, and for the purpose of this study we considered HD as a rough indicator of the beginning of anthesis period. The following temperature-derived variables were calculated for anthesis (A) as well the as for grain filling (GF) growth stage as described above. The temperature-derived variables used to quantify the duration and intensity of heat stress were: average maximum temperature (AMT), number of days with temperature > 30°C (NDTH30), number of days with temperature > 35°C (NDTH35) and heat degree days (HDD) (**Table 1**). HDD was estimated by adapting the equation of (Liu et al., 2016) (Equation 3).

**Table 1:** Weather variables calculated independently for each single plot to determine the heat stress experienced by plants from yield trials conducted under in Non-Stressed control and late planted Heat Stressed conditions involving the UNIBO-Durum Diversity Panel evaluated at CENEB-Cd. Obregon, Mexico, in 2018 and 2019.

Growth stage	Range of degree days
Anthesis (A)	300°C days before to 100°C post anthesis
Grain filling (GF)	100°C days to 600°C post anthesis

Weather variables^1^	Description
AMT_A and AMT_GF	Average maximum temperature (°C);
NDTH30_A and NDTH30_GF	Number of days with temperature > 30°C
NDTH35_A and NDTH30_GF HDD_A and HDD_GF	Number of days with temperature > 35°C Heat stress Degree Days °C

^1^_A and _GF: indicate Anthesis and Grain Filling, respectively.


(3) HDD=∑ (Tmax−Th)

Where: HDD is the accumulated heat degree days in °C; Tmax is the maximum daily temperature in °C; Th is the temperature threshold for heat stress. Considering the results obtained from the literature (Farooq et al., 2011; Liu et al., 2016, Liu et al, 2016; Telfer et al., 2018) we defined 30°C as the temperature threshold for heat stress.

### 2.5 Statistical analyses

#### 2.5.1 Data analyses

Analyses were performed separately for the different treatments, control (non-stress, NS) and heat-stress (HS), in two crop seasons (2018 and 2019) as well as jointly. The best linear unbiased estimates (BLUEs) for each trait in each treatment and year were calculated using the following mixed model (Equation 4):


(4) $$y_{ijkl}=\mu +{ɡ}_{i}+t_{j}+s_{k\left(j\right)}+r_{l\left(jk\right)}+{\left(ɡ\times t\right)}_{ij}+{\left(ɡ\times s\right)}_{ik\left(j\right)}+{\left(ɡ\times t\times s\right)}_{ijk\left(j\right)}+\varepsilon_{ijkl}$$

where *y_ijkl_* is the observed trait, *μ* is the overall mean, *g_i_* is the fixed effect of the genotype, *t_j_* is the fixed effect of the treatment, *s_k_*_(_ *_j_*_)_ is the random effect of the season (year) within the treatment, *r_l_*_(_ *_jk_*_)_ is the random effect of the replicate within the treatment, and the year and *ϵ_ijkl_* is the residual assumed to be normally and independently distributed (0, σ^2^). BLUEs were calculated using the *lmer4* package (Bates et al., 2015).

The statistical analysis was also performed within treatments with the following linear model (Equation 5):


(5) $$y_{ikl}=\mu +\;{ɡ}_{i}+\;s_{k}+r_{l\left(k\right)}+{\left(ɡ\times s\right)}_{ik}+\varepsilon_{ikl}$$

Broad-sense heritability for each treatment as well as crop season was calculated with the equation (Equation 6) using the function repeatability of the package repeatability (Wolak et al, 2012) in R software.


(6) $$h^{2}=\frac{\sigma_{ɡ}^{2}}{\sigma_{ɡ}^{2}+\;\frac{\sigma_{e}^{2}}{r}}$$

Where: $\;h^{2}$ is the broad-sense heritability, $\sigma_{g}^{2}$ and $\sigma_{e}^{2}$ are the genotype and error variance, respectively, and r is the number of replicates.

For the combined analysis, broad-sense heritability was calculated as (Equation 7) using the same function and software previously described.


(7) $$h^{2}=\frac{\sigma_{ɡ}^{2}}{\sigma_{ɡ}^{2}+\;\frac{\sigma_{ɡs}^{2}}{s}+\;\frac{\sigma_{e}^{2}}{sr}}$$

Where the new term $\sigma_{gs}^{2}\;$ is the genotype *×* season interaction and s is the number of seasons (years) in the experiment.

#### 2.5.2 Relationships among traits and structural equation models

Relationships among phenotypic traits were studied within each environmental condition (treatment), *i.e.*, control (non-stress, NS) and heat-stress (HS) treatments. The BLUEs were then used in all the subsequent analyses as adjusted phenotypic means. The relationships among all variables, *i.e.*, both plant traits and environmental covariates, were first evaluated by using Pearson phenotypic correlation calculated within treatment. To investigate the role of correlated traits in determining the final yield in the NS and in the HS environment, *i.e.*, to choose the most important traits to be included in a multi-trait model, the stepwise regression and LASSO (least absolute shrinkage and selection operator) (Tibshirani, 1996, Tibshirani, 2011) allowed us to select a subset of variables according to the results obtained and to previous knowledge. In addition, structural equation modeling (Gleason et al., 2019) was used to represent the relative importance of the yield component under NS and HS conditions. The final set of variables was combined into a SEM, where traits were treated as predictors (exogenous) or responses (endogenous) in a system of simultaneous equations, hence allowing us to establish functional (causal) links between phenotypes. The initial phenotypic SEM model was tested and then subjected to optimization by removing non-significant paths, one path at a time, in model testing and selection (He et al., 2021). For individual traits in the SEM the R^2^ statistic was calculated. Analyses were performed using the R package *lavaan* (Rosseel, 2012). Finally, based on SEM path coefficients, the net effects for each trait were estimated for both NS and HS environments.

In addition, the causal genotypic effects of traits on yield were investigated using Genetic-SEM (GSEM), as proposed by Kruijer et al. (2020) and implemented in the R package *pcgen*. The GSEM model was investigated by using the BLUEs from the two years for each stress treatment and assuming a threshold alpha at P = 0.01, obtained by bootstrapping. Only traits with direct or indirect genotypic effect on yield were retained in the final model and proposed as informative for prediction.

#### 2.5.3 Identification of sources of genetic tolerance to heat stress

Genotypes considered to be possible sources of genetic tolerance to heat stress were identified as those better performing under high temperature and/or those that registered the smallest decline from the NS to the HS treatment. Moreover, in the attempt to take into account the different phenology observed in the present study, performances were estimated as marginal means after adjusting for HD as covariate. We then fitted the BLUE data of HS and NS in the two seasons to a linear model including genotypes and HD as GDD as covariate, and calculating the marginal means with the package *emmeans* in R (Lenth, 2024).

## 3 Results

### 3.1 Effect of the growing conditions

The first aim of this study was to evaluate the effect of increased temperatures on morpho-physiological traits relevant to grain yield and yield components, and their relationship with environmental parameters, using a panel of durum wheat of diverse origins. The elite durum panel previously assembled at the University of Bologna (Maccaferri et al., 2005, Maccaferri et al., 2011) was evaluated in Obregon, Mexico, under field conditions in two contrasting environments, namely, timely-sown, optimal growth and management conditions (control or not stressed, NS) and late-sown with otherwise optimal management conditions, simulating a scenario of long exposure to supra-optimal temperatures and heat stress (HS) during all growing stages.

**Figure 1:**
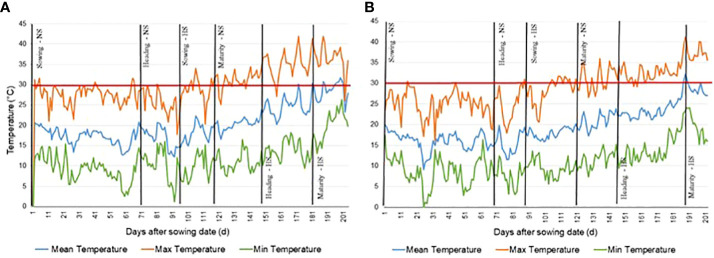
Maximum, minimum, and mean temperature near testing site of CENEB-Ciudad Obregon, Mexico from the sowing date of Non Stressed (NS) control experiment to the maturity date of the Heat Stressed (HS) experiment during the wheat growing season. **(A)** 2017/2018 growing season. **(B)** 2018/2019 growing season.

The difference in GDD and HDD accumulation across two consecutive crop seasons were considered. **Figure 1** reports the minimum, maximum and mean temperature during both crop seasons and shows that in 2018 the maximum temperature frequently, and right around heading, reached levels above 35°C during the grain filling period (GFP) in the HS experiment where values > 40°C were recorded in several days. This was the most important factor influencing the higher accumulation of HDD in 2018. On the other hand, the maximum temperature did not reach 35°C until the end of the plant growth cycle in 2019. The difference in HDD is thus one of the most important environmental parameters, accounting for the differences in phenotypic values for all traits in crop season 2018 when compared with the 2019 crop season. Plants evaluated over 2018 and 2019 accumulated different amounts of GDD and HDD to reach HD and DTM phenological growth stage (**Table 2**).

**Table 2:** Growing degree days (GDD) and heat degree days (HDD) accumulated up to heading (HD) and maturity (DTM) by plants from yield trials conducted under in Non-Stressed (NS) control and late planted Heat Stressed (HD) conditions involving the UNIBO-Durum Diversity Panel evaluated at CENEB-Cd. Obregon, Mexico, in 2018 and 2019.

Evaluation condition (Treatment)	Trait	GDD (d)			HDD (d)			Difference in GDD between years	Difference in HDD between years
		2018	2019	Mean	2018	2019	Mean	%	%
NS	HD	1481.7	1360.2	1421.0	6.6	0.8	3.7	8.2	87.9
	DTM	2280.0	2296.6	2288.3	26.1	25.9	26.0	0.7	0.8
HS	HD	1251.0	1202.5	1226.8	93.0	62.5	77.8	3.9	32.8
	DTM	1912.5	1896.9	1904.7	230.5	131.8	181.2	0.8	42.8

Slightly higher levels of GDD were observed in the NS treatment than in the HS, for both time to heading and time to maturity. However, the late sowing-generated heat stress did not have a very pronounced effect on GDD, with about 15.0% of difference in GDD between the timely and late planting treatments. The HDD was lower in NS than in HS for both HD and DTM, with late sowing dramatically increasing HDD, especially when considering DTM. This result is obviously consistent with a higher level of temperature stress in the late sowing trials. The level of heat stress was slightly different in the two years, more pronounced in 2018 than in 2019. HDD accumulation was more pronounced in 2018 with 32.8% more HDD from sowing to HD and 42.8% from sowing to DTM (**Table 2**). However, since the mean square for the year *×* treatment interaction was consistently lower than those for year and treatment for all traits (**Table 3**), we hereafter report the analyses combined over years.

**Table 3:** Summary, two-years combined, ANOVA for days to heading (HD), days to maturity (DTM), grain filling period (GFP), plant height (PH), grain yield (GY), thousand kernel weight (TKW), test weight (TWT), spikes per linear meter (SPM), spikelets per spike (SKT), kernel number per spike (KNS), kernel weight per spike (KWS), kernel length (KLE), kernel width (KWI), normalized difference vegetation index (NDVI), and area under heat stress progress curve for NDVI (IT_NDVI) collected on plants from yield trials conducted under in Non-Stressed control and late planted Heat Stressed conditions involving the UNIBO-Durum Diversity Panel evaluated at CENEB-Cd. Obregon, Mexico, in 2018 and 2019.

Mean squares							
Trait	Year	Treatment	Genotype	Gen×Treat^1^	Year×Gen	Year×Treat	Year×Gen×Treat
HD	24.0***	165203***	176.0***	31.0***	7.0***	467.0***	5.0***
DTM	11657***	580606***	119.0***	24.0***	6.0***	1771***	4.0***
GFP	10367***	124619***	23.0***	17.0***	7.0***	480.0***	8.0***
PH	12087***	263490***	481.0***	103.0***	29.0***	6.0ns	19.0***
GY	456.0***	4780***	3.1***	0.8***	0.7**	49.0***	0.5***
TKW	12724***	26371***	156.0***	35.0***	8.0***	1356***	5.0***
TWT	32.0***	1968***	19.0***	3.0***	1.0***	96.6***	0.67***
SPM	13848***	329166***	755.0***	298.0***	228.0***	1664***	166.0**
SKT	336.0***	2503***	11.0***	4.0***	2.0***	216.0***	1.0***
KNS	14005***	94141***	279.0***	54.0***	32.0***	2539***	23.0***
KWS	164.0***	594.0***	0.7***	0.2.0***	0.1***	3.0***	0.1*
KLE	0.3***	17.0***	0.5***	0.03***	0.01***	8.3***	0.01***
KWI	3.0***	27.0***	0.1***	0.02***	0.005***	0.003ns	0.004**
NDVI	0.57***	57240***	0.017***	0.009***	0.004***	0.022***	0.003***
IT_NDVI	6639333***	12181038***	4596***	2137***	865.0***	3178566***	905.0***

^1^ Gen×treat, genotype per treatment interaction; Year×Treat, year per treatment interaction, and Year×Gen×Treat, year per genotype per treatment interaction.

***P-v alue< 0.001, **P-value< 0.01, *P-value< 0.05, ns, not significant at P< 0.05.

### 3.2 Phenotypic field response to heat stress

The analysis of variance (ANOVA) showed significant effects for all single factors (year, treatment, and genotype) as well as for all interactions (genotype x treatment, year x genotype, year x treatment, year x genotype x treatment) for almost all traits **Table 3**. Summary statistics combined over years are reported in **Table 4**, while the results for a single year and a single treatment are reported in **Supplementary Tables 4**, **5**. The heat stress experienced by plants during both crop seasons significantly reduced the phenotypic value for all traits. The effect of heat stress was particularly pronounced for GY (-56.6%), NDVI as a proxy measurement for total biomass (-51.3%) and KWS (-40.5%). All yield components were affected, from SPM (-23.9%) through KNS (-29.2%) to TKW (-14.7%). It also substantially affected phenological traits with reductions of 25.8% for HD, 30.6% for DTM and 38.7% for GFP. TWT (-2.8%) and other kernel shape measurements (KLE, -2.7% and KWI, -7.6%) were the least affected.

**Table 4:** Two-years combined summary statistics for days to heading (HD), days to maturity (DTM), grain filling period (GFP), plant height (PH), grain yield (GY), thousand kernel weight (TKW), test weight (TWT), spikes per linear meter (SPM), spikelets per spike (SKT), kernel number per spike (KNS), kernel weight per spike (KWS), kernel length (KLE), kernel width (KWI), normalized difference vegetation index (NDVI), and area under heat stress progress curve for NDVI (IT_NDVI), observed in a yield trials conducted under in Non-Stressed control and late planted Heat Stressed conditions involving the UNIBO-Durum Diversity Panel evaluated at CENEB-Cd.

Trait	Non Stressed					Heat Stressed					% of change ^4^
	Mean	Range	Std ^1^	CV (%) ^2^	*h^2^* ^3^	Mean	Range	Std	CV (%)	*h^2^*	
HD (d)	80.3	67.0–108.0	6.60	2.71	0.97	59.6	52.0–84.0	3.84	2.54	0.95	25.8
DTM (d)	126.9	116.0–147.0	6.51	1.80	0.74	88.1	80.0–104.0	4.19	2.13	0.86	30.6
GFP (d)	46.8	32.0–59.0	4.62	9,64	0.21	28.7	18.0–40.0	3.26	10.44	0.43	38.7
PH (cm)	83.6	60.0–150.0	11.17	5.23	0.93	57.5	30.0–105.0	8.38	7.68	0.82	31.2
GY (t/ha)	6.2	2.9–10.3	1.26	13.13	0.34	2.7	0.2–5.4	0.94	21.90	0.72	56.6
TKW (g)	56.1	37.1–71.6	5.92	3.74	0.92	47.8	30.7–65.7	6.21	4.56	0.70	14.7
TWT (kg/hL)	80.4	70.2–83.9	1.76	0.84	0.96	78.1	70.7–82.3	1.81	1.13	0.91	2.8
SPM (n)	122.0	82.0–190.0	18.57	11.66	0.65	92.8	48.0–138.0	15.48	13.44	0.53	23.9
SKT (n)	18.4	14.2–28.0	2.15	7.08	0.74	15.9	12.2–20.0	1.29	5.55	0.82	13.8
KNS (n)	53.7	31.2–84.5	9.49	9.16	0.77	38.0	14.7–65.2	6.75	10.88	0.83	29.2
KWS (g)	3.1	1.6–4.8	0.59	9.40	0.55	1.8	0.5–3.3	0.42	11.76	0.50	40.5
NDVI	7.6	6.6–8.5	0.28	1.14	0.95	7.4	6.4–8.2	0.29	1.51	0.91	2.7
IT_NDVI	3.5	3.1–3.9	0.15	1.59	0.91	3.2	2.7–3.8	0.15	2.02	0.89	7.6
KLE (mm)	0.8	0.546–0.870	0.04	4.92	0.64	0.4	0.163–0.727	0.10	20.44	0.73	51.3
KWI (mm)	435.9	208.2–598.6	114.12	29.81	0.20	257.3	122.6–418.0	52.49	16.58	0.64	41.0

*1* std, standard deviation. *2* CV (%), coefficient of variation. *3* h2, broad-sense heritability. *4*% of change, percentage of variation in heat stress treatment relative to value under optimal conditions.

### 3.3 Relationship among traits and modeling of the stress response

Phenotypic correlations among plant traits in NS and HS treatments are presented numerically in **Table 5** and graphically in **Supplementary Figure 1.** The analysis of relationships among traits revealed that the phenological parameters (HD, DTM) were negatively, but weakly, correlated with GY in both testing conditions, suggesting a very weak trend that earlier genotypes were, to some extent, higher yielding than later ones under optimal conditions, a trend that maintained its small magnitude under HS. On the other hand, the association between phenological parameters and the most important kernel characteristics (TKW and TWT) indicated a different dynamic. Correlations of intermediate magnitude indicated that genotypes heading or maturing earlier generally had larger kernels with greater test weights under non-stressed conditions. These relationships changed under heat stress, with no significant association observed between HD and TKW and a reversed positive, albeit very weak, association between DTM and TKW. The intermediate strong associations between phenological parameters and TWT observed under NS conditions were maintained under HS but with an observable or substantial decrease in magnitude. There was no association detected between phenological parameters and spike number (SPM) under NS conditions, while a weak negative association was detected between HD and SPM under HS suggesting, to a small extent, that earlier genotypes tended to produce more spikes than later ones. Both phenological parameters were strongly and positively associated with spike size, as determined by SKT, under NS and, to a somewhat smaller extent, under HS conditions, indicating a strong and across-environment trend that later heading and/or maturing genotypes tended to produce more spikelets per spikes and therefore larger spikes, regardless of heat stress. The relationships between phenological traits and biomass indicators (NDVI and IT-NDVI) were among the strongest observed under NS and, while somewhat decreased in magnitude under HS for HD, they were maintained, indicating that later and/or later-maturing genotypes tended to produce more biomass in normal conditions as well as under heat.

**Table 5:** Pearson correlation coefficient between traits observed under in Non-Stressed Control (below diagonal) and under late planted Heat Stress (above diagonal) conditions in a durum diversity panel (N=187) evaluated at CENEB-Cd Obregon, Mexico in 2018 and 2019 (values are 2-years average BLUEs). Traits: days to heading date (HD), days to maturity (DTM), grain filling period (GFP), plant height (PH), grain yield (GY), thousand kernel weight (TKW), test weight (TWT), spikes per linear meter (SPM), spikelets per spike (SKT), kernel number per spike (KNS), kernel weight per spike (KWS), kernel length (KLE), kernel width (KWI), normalized difference vegetation index (NDVI), and area under heat stress progress curve for NDVI (IT_NDVI).

Heat Stressed																
		HD	DTM	GFP	PH	GY	TKW	TWT	SPM	SKT	KNS	KWS	KLE	KWI	NDVI	IT_ NDVI
Non Stressed	**HD**		0.82**	-0.03ns	-0.03ns	-0.29**	-0.04ns	-0.35**	-0.23**	0.54**	-0.01ns	-0.10ns	-0.04ns	-0.01ns	0.56**	0.50**
	**DTM**	0.93**		0.54**	0.27**	-0.06ns	0.21**	-0.22**	-0.10ns	0.57**	0.08ns	0.13ns	0.02ns	0.22**	0.77**	0.71**
	**GFP**	-0.69**	-0.37**		0.51**	0.32**	0.43**	0.14ns	0.15 *	0.22**	0.17 *	0.36**	0.09ns	0.42**	0.53**	0.52**
	**PH**	0.45**	0.44**	-0.27**		0.51**	0.24**	0.36**	0.40**	0.13ns	0.26**	0.37**	-0.03ns	0.18 *	0.47**	0.47**
	**GY**	-0.20**	-0.25**	0.02ns	-0.05ns		0.29**	0.49**	0.58**	-0.05ns	0.49**	0.64**	0.19 *	0.09ns	0.36**	0.44**
	**TKW**	-0.42**	-0.44**	0.21**	-0.25**	0.18**		0.07ns	0.02ns	-0.02ns	-0.23 *	0.31**	0.65**	0.82**	0.32**	0.33**
	**TWT**	-0.49**	-0.51**	0.24**	-0.09ns	0.43**	0.26**		0.47**	-0.12ns	0.30**	0.36**	-0.14ns	-0.11ns	0.11ns	0.12ns
	**SPM**	0.12ns	0.10ns	-0.10ns	0.49**	0.11ns	-0.28**	0.01ns		-0.10ns	0.16 *	0.18 *	0.02ns	-0.12ns	0.19 *	0.28**
	**SKT**	0.75**	0.77**	-0.37**	0.37**	-0.16**	-0.45**	-0.45**	-0.01ns		0.31**	0.27**	-0.07ns	-0.02ns	0.45**	0.42**
	**KNS**	0.19**	0.17 *	-0.15 *	0.02ns	0.19**	-0.46**	0.06ns	-0.31**	0.35**		0.82**	-0.23 *	-0.24 *	0.25**	0.25**
	**KWS**	-0.13ns	-0.17 *	0.01ns	-0.16 *	0.36**	0.28**	0.28**	-0.55**	0.02ns	0.69**		0.16 *	0.18 *	0.36**	0.38**
	**KLE**	-0.09ns	-0.17 *	-0.10ns	-0.17 *	0.06ns	0.61**	-0.21**	-0.13ns	-0.19 *	-0.32 *	0.14 *		0.32**	0.11ns	0.17 *
	**KWI**	-0.38**	-0.36**	0.24**	-0.18 *	0.08ns	0.87**	0.24**	-0.29**	-0.37**	-0.41**	0.22**	0.29**		0.18 *	0.20**
	**NDVI**	0.75**	0.73**	-0.45**	0.15 *	0.06ns	-0.26**	-0.29**	0.02ns	0.53**	0.16 *	-0.06ns	-0.02ns	-0.28 *		0.95**
	**IT_NDVI**	0.79**	0.78**	-0.45**	0.21**	0.04ns	-0.28**	-0.28**	0.01ns	0.56**	0.20**	-0.02ns	-0.07ns	-0.28**	0.92**	

*p<0.1; **p<0.05; ***p<0.01; ns, non significant.

To adjust for differences in phenology among the diverse genotypes included in the panel, we performed again the analysis for all traits using HD as covariate. To get HD-unbiased indications of trends, the adjusted data (marginal means) were again inspected for correlation patterns (**Supplementary Figure 2**). The adjusted correlation data clearly pointed out that under non-stressed conditions GY was primarily and positively associated with spike size and fertility (GY-SKT, *r* = 0.79; GY-KNS, *r* = 0.77), with kernel size (GY-TKW, *r* = 0.25; GY-KWS, *r* = 0.85) and moderately but negatively associated with spike number (GY-SPM, *r* = -0.29). This indicated that with bigger and more fertile spikes, producing larger grains and generally having less spikes per area, tended to be those which yielded the most under optimal conditions. Under the heat stress condition of this experiment, the association between GY spike size/fertility (SKT, KNS) or kernel size (KWS, TKW), was either lower or similar in magnitude (GY-SKT, *r* = 0.37; GY-KNS, *r* = 0.59; GY-KWS, *r* = 0.77; and GY-TKW, r = 0.59). The association with spike number was significantly modified under heat becoming moderately positive (GY-SPM, *r* = 0.36);. The adjustment for HD covariate made a considerable difference in detecting the association between GY and biomass indicators (NDVI and IT_NDVI) under NS conditions. Although no significant correlation was initially found between GY and biomass indicators under optimal conditions using the non-adjusted data, the adjustment revealed a very strong positive association between these traits. This indicates that, with similar phenology, genotypes with higher biomass production tend to have higher yields (GY-NDVI, r = 0.88; IT_NDVI, r = 0.87). Under heat stress, the adjustment transformed weak positive correlations into very strong ones (GY-NDVI, r = 0.77; IT_NDVI, r = 0.78), confirming that the relationship between GY and biomass indicators persists even under thermal stress. NDVI measured at medium milk-soft dough stage under non-stressed conditions was only weakly associated with grain weight (TKW-NDVI, *r* = 0.25) but highly correlated with spike fertility (KNS-NDVI, *r* = 0.78). Under HS however, the biomass indicator became strongly associated with grain weight (TKW-NDVI, *r* = 0.74), maintaining a relatively robust association with spike fertility parameters (SKT-NDVI, *r* = 0.49; KNS-NDVI, *r* = 0.44).

**Table 6** reports the correlations between original non-adjusted phenotypic traits and weather variables. Under both NS and HS conditions, GY and temperature-related variables showed always negative correlations, but very weak, if at all significant. The associations involving PH or TKW were moderate under NS conditions and non-significant under HS. SPM, weakly correlated with temperature variables in grain fill under NS, became slightly negatively correlated under heat. All other yield components showed generally low correlations with weather variables, especially under HS. TWT was variably correlated with temperature variables under NS, especially to those indicative of high temperatures during grain fill, but showed correlation generally non-significant in HS condition. In terms of the biomass indicators, NDVI and IT_NDVI showed the highest magnitude associations with temperature derived variables, more so with those related to temperatures during grain fill under non-stressed conditions and these associations remained significant under heat stress, albeit with a reduced strength.

**Table 6:** Pearson correlation coefficient between plant traits (see text for description) measured in yield trials conducted under in Non-Stressed control and late planted Heat Stressed conditions involving the UNIBO-Durum Diversity Panel evaluated at CENEB-Cd. Obregon, Mexico, in 2018 and 2019 and weather variables: average maximum temperature at anthesis (AMT_A) and grain filling period (AMT_GF), number of days with temperature higher than 30°C at anthesis (NDTH30_A) and grain filling period (NDTH30GF), number of days with temperature higher than 35°C at anthesis (NDTH35_A) and grain filling period (NDTH35_GF), and heat degree days at anthesis (HDD_A) and grain filling period (HDD_GF).

	AMT_ A	AMT_ GF	NDTH 30_A	NDTH 35_A	NDTH 30_GF	NDTH 35_GF	HDD_ A	HDD_ GF
Non Stressed								
**HD**	0.39**	0.73**	0.55**		0.97**	0.73**	0.24**	0.97**
**DTM**	0.33**	0.65**	0.51**		0.90**	0.69**	0.21**	0.90**
**GFP**	-0.33**	-0.54**	-0.39**		-0.67**	-0.48**	-0.18 *	-0.65**
**PH**	0.20**	0.29**	0.35**		0.48**	0.24**	0.21**	0.42**
**GY**	-0.08ns	-0.18 *	-0.16 *		-0.22**	-0.18 *	-0.22**	-0.20**
**TKW**	-0.24**	-0.32**	-0.28**		-0.43**	-0.35**	-0.10ns	-0.43**
**TWT**	-0.25**	-0.37**	-0.29**		-0.49**	-0.49**	-0.25**	-0.51**
**SPM**	0.11ns	0.10ns	0.11ns		0.17 *	0.22**	0.10ns	0.17 *
**SKT**	0.33**	0.53**	0.51**		0.75**	0.59**	0.27**	0.75**
**KNS**	0.16 *	0.13ns	0.18 *		0.14ns	-0.02ns	-0.05ns	0.13ns
**KWS**	-0.05ns	-0.11ns	-0.04ns		-0.18 *	-0.30**	-0.15 *	-0.21**
**KLE**	0.03ns	-0.12ns	0.01ns		-0.10ns	-0.09ns	0.11ns	-0.12ns
**KWI**	-0.25**	-0.28**	-0.28**		-0.38**	-0.30**	-0.12ns	-0.38**
**NDVI**	0.27**	0.54**	0.35**		0.71**	0.54**	0.07ns	0.71**
**IT_NDVI**	0.27**	0.60**	0.36**		0.75**	0.54**	0.05ns	0.74**
Heat Stressed								
**HD**	0.72**	0.94**	0.32**	0.79**	0.61**	0.25**	0.60**	0.95**
**DTM**	0.59**	0.80**	0.25**	0.65**	0.51**	0.22**	0.50**	0.81**
**GFP**	-0.04ns	0.01ns	-0.03ns	-0.02ns	0.01ns	0.02ns	-0.01ns	0.02ns
**PH**	0.01ns	0.02ns	0.04ns	-0.06ns	0.07ns	0.12ns	0.05ns	-0.01ns
**GY**	-0.22**	-0.24**	-0.10ns	-0.28**	-0.06ns	0.03ns	-0.15 *	-0.24**
**TKW**	-0.03ns	0.01ns	-0.04ns	-0.05ns	0.05ns	0.11ns	0.02ns	-0.02ns
**TWT**	-0.23**	-0.32**	-0.01ns	-0.35**	-0.10ns	-0.09ns	-0.19 *	-0.35**
**SPM**	-0.12ns	-0.21**	0.02ns	-0.23**	-0.09ns	0.02ns	-0.09ns	-0.21**
**SKT**	0.32**	0.53**	0.20**	0.35**	0.34**	0.14ns	0.24**	0.51**
**KNS**	-0.05ns	-0.02ns	0.12ns	-0.12ns	0.05ns	-0.05ns	-0.10ns	-0.02ns
**KWS**	-0.11ns	-0.07ns	0.04ns	-0.16**	0.05ns	-0.01ns	-0.11ns	-0.09ns
**KLE**	-0.02ns	-0.02ns	-0.01ns	-0.08ns	0.10ns	0.06ns	0.01ns	-0.03ns
**KWI**	0.01ns	0.01ns	-0.02ns	0.01ns	0.01ns	0.06ns	0.05ns	-0.01ns
**NDVI**	0.41**	0.55**	0.25**	0.38**	0.43**	0.16 *	0.35**	0.55**
**IT_NDVI**	0.37**	0.49**	0.22**	0.33**	0.37**	0.18 *	0.33**	0.50**

*p<0.1; **p<0.05; ***p<0.01.

After considering direct or simple correlations between variables and to better understand the complex picture of the factors involved and their interactions, multiple models were considered. Stepwise regression and LASSO were used to evaluate which traits were the best predictors for GY under the two testing conditions and the best set of variables for the comparison between NS and HS environments (**Table 7**). Results from the stepwise regression showed that in non-stress conditions, 11 out of 22 traits were retained, with the Adj. R^2^. *i.e*., the R^2^ adjusted for the number of traits, increasing from 0.441 to 0.453 while under heat stress conditions, 12 of the 23 traits resulted in an increased Adj. R^2^ from 0.761 to 0.771. With LASSO lambda min, 12 traits were retained under normal conditions, with Adj. R^2^ 0.of 430 while 18 traits were left in the set selected for the HS treatment, with an Adj. R^2^ of 0.766. Stepwise regression and LASSO revealed that three plant variables most affecting GY under both testing conditions were DTM (negatively), SPM (positively) and biomass (NDVI or IT_NDVI, both positively) and the temperature-related variables with most impact on GY were AMTGF (negatively) and HDD_GF (positively). These models also revealed several variables which contributed to GY in only one or the other testing environment: TWT and KWS only under NS conditions while PH, TKW and KNS exclusively under HS conditions. In terms of the temperature-related variables affecting GY only in one testing environment, NDTH30A was detected by both stepwise regression and LASSO as negatively affecting performance under heat stress.

**Table 7:** Multiple regression models fitted in Non Stressed or Heat Stressed treatments from yield trials involving the UNIBO-Durum Diversity Panel evaluated at CENEB-Cd. Obregon, Mexico, in 2018 and 2019, with the dependent variable grain yield (GY).

	Dependent variable GY (t ha^-1^)					
	Non Stressed			Heat Stressed		
Independable variable	No selection	Stepwise selection	LASSO selection	No selection	Stepwise selection	LASSO selection
HD	1.554	1.531	-0.017	-0.018		-0.014
	(0.962)	(0.928)	(0.021)	(0.494)		(0.062)
DTM	-1.683*	-1.639*	-0.046*	-0.059	-0.079***	-0.069***
	(0.964)	(0.929)	(0.025)	(0.479)	(0.018)	(0.02)
GFP	1.630*	1.599*		-0.01		
	(0.966)	(0.931)		(0.478)		
PH	0.001			0.016***	0.017***	0.016***
	(0.006)			(0.006)	(0.005)	(0.005)
TKW	0.026		0.004	0.042*	0.061***	0.043*
	(0.034)		(0.012)	(0.024)	(0.011)	(0.023)
TWT	0.079**	0.083***	0.073**	0.020		0.021
	(0.039)	(0.03)	(0.036)	(0.025)		(0.025)
SPM	0.025***	0.024***	0.025***	0.018***	0.019***	0.018***
	(0.005)	(0.004)	(0.004)	(0.004)	(0.003)	(0.003)
SKT	0.056			-0.055	-0.056*	-0.054
	(0.046)			(0.034)	(0.032)	(0.033)
KNS	-0.007			0.053***	0.056***	0.053***
	(0.023)			(0.019)	(0.005)	(0.018)
KWS	1.027**	1.024***	0.913***	0.074		0.054
	(0.402)	(0.144)	(0.153)	(0.396)		(0.381)
KLE	-0.084		0.064	0.17		0.17
	(0.323)		(0.244)	(0.204)		(0.194)
KWI	-0.77			-0.279	-0.582*	-0.282
	(0.864)			(0.491)	(0.351)	(0.463)
NDVI	7.242**	11.004***	7.472**	0.102		
	(3.545)	(2.024)	(3.456)	(1.441)		
IT_NDVI	0.009		0.008	0.008***	0.008***	0.008***
	(0.006)		(0.006)	(0.003)	(0.001)	(0.001)
AMTA	0.424*	0.319*		-0.292		-0.288
	(0.227)	(0.186)		(0.795)		(0.781)
AMTGF	-0.101*	-0.089*	-0.05	-1.157**	-1.122**	-1.183**
	(0.059)	(0.052)	(0.048)	(0.579)	(0.461)	(0.564)
NDTH30A	-0.062		0.082	-0.344**	-0.340***	-0.310***
	(0.228)		(0.2)	(0.154)	(0.078)	(0.111)
NDTH35A				-0.063		
				(0.194)		
NDTH30GF	-0.128			-0.054		-0.025
	(0.171)			(0.154)		(0.119)
NDTH35GF	-0.776			0.068		0.088
	(0.74)			(0.142)		(0.12)
HDD_A	-0.099	-0.148**	-0.112	0.058	0.026**	0.049
	(0.148)	(0.07)	(0.121)	(0.054)	(0.012)	(0.046)
HDD_GF	0.174*	0.084**		0.074**	0.066**	0.074**
	(0.092)	(0.04)		(0.037)	(0.031)	(0.036)
Constant	-10.143	-10.822**	-6.548	45.143*	38.963***	45.168*
	(8.197)	(5.175)	(4.953)	(25.03)	(13.396)	(24.556)
Observations	183	183	183	181	181	181
R^2^	0.505	0.486	0.467	0.790	0.787	0.790
Adjusted R^2^	0.441	0.453	0.430	0.761	0.771	0.766
Residual Std. Error	0.544	0.538	0.549	0.348	0.340	0.345
F Statistic	7.826***	14.690***	12.429***	27.098***	51.645***	31.945***
	(df = 21; 161)	(df = 11; 171)	(df = 12; 170)	(df = 22; 158)	(df = 12; 168)	(df = 19; 161)

*p<0.1; **p<0.05; ***p<0.01. Models were defined as No selection, with all plant and environmental independent variables; Stepwise selection, with independent variables selected with stepwise procedure; LASSO selection, with dependent variable were selected by the least absolute shrinkage and selection operator method (Tibshirani, 1996, Tibshirani, 2011). (SE in parentheses).

The final network was translated into a SEM, where phenotypes can be treated both as predictor (exogenous) and response (endogenous) in a system of simultaneous equations, hence allowing to postulate functional (causal) links between traits (**Supplementary Figure 3**). SEM coefficients were estimated and standardized path coefficients are represented by arrows, with the green indicating a positive relationship while the red ones indicate a negative relationship, the thickness being proportional to the relative coefficient size. The results of the analysis are reported in **Supplementary Table 6**. The final set of variables reached an R^2^ 0.423 in NS and 0.712 under HS conditions. These values are not much lower than those obtained with the whole model. In the best-fitted model, in NS conditions, GY appeared to be directly related to TKW, TWT, SPM, KNS, and HDD did not show significant adverse effects regardless of when, up to anthesis or during grain filling, it was accumulated. Under HS conditions, the plant variables PH, TKW, SPM, KNS and IT_NDVI were directly related to GY while HDD during grain fill showed a negative effect on GY.

**Figure 2:**
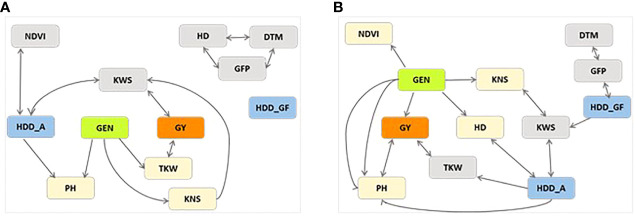
GSEM estimated networks with P< 0.01 for traits days to heading (HD), days to maturity (DTM), grain filling period (GFP), plant height (PH), grain yield (GY), thousand kernel weight (TKW), kernel number per spike (KNS), kernel weight per spike (KWS), normalized difference vegetation index (NDVI) and heat degree days at anthesis (HDD_A) and at grain filling period (HDD_GF), collected on plants from yield trials involving the UNIBO-Durum Diversity Panel evaluated at CENEB-Cd. Obregon, Mexico, in 2018 and 2019. Besides GEN representing the genetic effect in green, GY is highlighted in orange and the other traits directly connected with GEN were highlighted in pale-orange. Weather variables were highlighted in blue. **(A)** Non Stressed control. **(B)** Heat Stressed.

The PCgen reconstruction of networks is presented in **Figure 2**. PCgen was applied to the BLUEs of the single year for each treatment rather than to single plot values, and for this reason the genetic relationship among traits is not affected by the residual genotype by year interaction. With this method, the genetic effects are incorporated in the network reconstruction and thus direct genetic effects can be detected. In timely-sown control conditions, only PH, TKW, KNS showed a direct genetic effect, while GY showed an indirect effect through TKW. Under HS conditions, a direct significant genetic effect was detected for HD, PH, NDVI, KNS, and GY. The other traits showed edges not significant at P 0.01 **(Supplementary Table 7**). The reconstruction of networks, reported in **Figure 2**, also includes the effects of HDD. In control conditions, the HDD_GF showed no connection with other traits, while HDD_A was connected with NDVI and KWS, even though not through genetic effects. Under stress conditions HDD_A was connected with HD, PH, TKW, and KWS. On the other hand, HDD_GF was in turn connected in a cluster with GFP-DTM and KWS, in accordance with what was already seen in SEM analysis.

### 3.4 Identification of sources of genetic tolerance to heat stress

The performances of varieties in NS and HS conditions, and the GY loss due to heat stress were considered in order to identify potential sources of tolerance to heat stress. Based on a significant confounding effect of phenology on yield performance in the different testing conditions, the performances of genotypes were also estimated as marginal means from the average response to HD (see the **Supplementary Tables 8** and **S9** for the detailed non adjusted or adjusted values based on HD covariate genotype values).

In reaction to heat stress, all varieties showed a reduction in HD, ranging from -3% to -23%. Groups of varieties already adapted to warm growing environments with relatively short growing cycle like the Desert Durums (Kofa, Kronos, WestBred881, Bravadur) and CIMMYT’80s cultivars (Altar 84, Iride, several CIMMYT lines and Spanish varieties) showed a reduction in HD generally below 10%. Conversely, Italian and, in part, ICARDA varieties and lines bred for longer growing cycles showed a more marked reduction in HD.

Based on the marginal means after adjustment for HD (**Supplementary Table 9**), the three variables that were most reduced by heat stress were GY, then biomass (NDVI) and then the variables related to spike fertility (KWS/KNS). Losses in GY ranged from -22.8% to -85.8%, with an average loss of -56.0%. In terms of biomass (NDVI), losses ranging from 28% to 77%, with an average of 52% were recorded. Losses in spike fertility were indicated by a loss in KWS ranging from 3% to 70%, averaging 39%. The varieties that showed lower-than-average yield losses were frequently found again among the Desert Durums, CIMMYT’80s and with some ICARDA temperate groups. When dissecting the overall grain yield loss at the level of the less complex grain yield components, we could identify varieties/varietal groups which responded to HS differently. Considering the major subpopulations/groups, Subpop 1 - ITALY-MEDITERRANEAN and Subpop 2 - ICARDA-DRYLAND were mainly affected for biomass production (IT_NDVI or NDVI) and therefore canopy development, growth, biomass accumulation, and photosynthetic capacity. The Italian varieties were less affected at the level of grain weight TKW and test weight TWT but with marked reductions observed in spike fertility. Subpop 3 - ICARDA-TEMPERATE, also including some Desert Durums, showed a wide range of responses with varieties more resilient in terms of their biomass production and spike fertility (SKT and KNS). These included the Desert Durums Bravadur, WB881, Produra, Kofa, and ICARDA (Cham1, Stojocri). Notably, Subpop 4 - CIMMYT’70 - ICARDA also showed also a wide range of responses with the absence of a clear trend. Subpop 5 - CIMMYT’80 included the best materials in terms of biomass production and fertile culms/spike density, as well as for maintenance of spike fertility.

## 4 Discussion

The frequency and severity of high-temperature stress have increased consistently in the past decade and are expected to reach worrisome levels soon (Asseng et al., 2017; Zhao et al., 2017; IPCC, 2023), which underlines the urgency for a better dissection of the genetic control of HS resilience in order to identify native haplotypes which may allow to mitigate the effects of heat stress in crops like durum wheat which is prevalently grown in relatively heat-prone regions such as the Mediterranean Basin (Kutiel, 2019), and areas already characterized by high heat stress such as Central and Peninsular India and Iran or west Africa such as Senegal valley (Sall et al., 2018). The heat stress intensity to which plants were exposed in this study proved to be very effective, as expected based on the average temperatures typical of the location and sowing times. The experimental location was chosen because it is highly productive when wheat is sown at optimum time, and with a major constraint due to high temperature associated to late sowing is applied. The present study was conducted over two years, with higher GY in 2019, due to less extreme temperatures during grain filling, both under control and late sowing treatment. Even with this difference between testing years, the heat stress to which the panel was subjected reduced GY by 57% on average, with a very wide range of yield losses observed overall, both within and among the different germplasm groups represented in this panel. Other authors (Sukumaran et al., 2018), working at the same location on durum wheat, have reported a higher reduction of GY (71.7%) due to HS. As an effect of the HS, the time to maturity was markedly shortened as well, in agreement with previous observations (Djanaguiraman et al., 2020). Temperature-related variables that affected yield under HS were generally those calculated based on temperatures at grain fill (post anthesis), confirming what has been observed in several small-grains species, that some of the most important yield-limiting effects of heat on the present durum panel were related to high temperatures from flowering through grain filling stages, which affects several reproductive and/or physiological processes (Cossani and Reynolds, 2012; Fabian et al., 2019), or grain metabolic pathways (Wahid et al., 2007; Farooq et al., 2011; Gautam et al., 2014; Sharma et al., 2017). However, HS prior to anthesis and grain fill is also critically detrimental, affecting final yield consequent to a reduced source capacity and/or biomass available to support grain filling, particularly in its final stages (Xu et al., 2022; Barratt et al, 2024; Sall et al., 2024). In the current study, a substantial reduction was observed in biomass accumulated up to shortly after anthesis (NDVI), averaging 52%, second only to the effect on final GY. For this trait as well, a wide range of losses due to heat were observed both between and within germplasm groups included in the panel.

Environmental variables were used as covariates when they were proven to affect genotypes’ performance. Inevitably, different lines were subjected to different conditions due to their specific phenology that, notably, was purposedly kept within a 1-week interval among the tested genotypes. Moreover, the effect of the stress treatment imposed by delayed sowing likely induced differential responses depending on the timing of the most limiting conditions, particularly whether they occurred before or after heading. For these reasons, to get a comprehensive picture of the inter-relations among several environmental parameters and with yield and the other traits, we undertook further analyses following three steps. First we applied stepwise regression and LASSO for reducing the confounding factors in the predictive model to keep only those which mostly affected GY. Based on this analysis, NDTH30_A and AMT_GF impacted significantly and negatively final yield to a higher extent as compared to the other environmental variables that showed some significant effects such as AMT_A, HDD_A, HDD_GF. Other environmental variables such as NDTH35_A, NDTH30_GF, and NDTH35_GF did not affect significantly GY. This means that the negative effects on GY are already, and mostly, determined by the cumulative number of days with temperatures passing the relatively mild sensitivity threshold of 30°C, rather than 35°C which corresponds to the typical heat stress wave, and are already determined between anthesis and grain filling, reviewed by Ullah et al. (2022) and Akter and Islam (2017).

Structural Equation Models (SEMs) have been used to study recursive and simultaneous relationships among phenotypes in multivariate systems such as multiple-trait models in quantitative genetics (Valente et al., 2010) to identify a network of correlated traits (He et al., 2021), together with genome-wide SNP profiles. As a second step toward the understanding of the response to HS, we employed SEM based on phenotypic and environmental variables. The possible use of SEMs to understand physiological causes of genotype by environment or management interaction was explored by Vargas et al. (2007), who concluded that the approach can facilitate the understanding of the effects of environmental covariates on yield performances or can be used to generate hypotheses (Valente et al., 2013). Under heat stress, SEM confirmed the role of HDD_A and HDD_GF in controlling NDVI (as NDVI and IT_NDVI), hence plant growth and biomass development, which in turn plays a key pivotal role in influencing of PH, KNS, TKW and finally GY, as recently pointed out by Kumar et al. (2023).

Finally, as the third step to improve the understanding of the multiple trait models, we considered one of the methods suggested to identify which variable most affects genotypes’ performance (Maathuis et al, 2009; Maathuis et al., 2010; Buhlmann et al, 2014; Buhlmann, 2020). Recently, GSEM has been proposed (Kruijer et al., 2020), *i.e*., the linear genetic structural equation models, which allows for the reconstruction of a causal model compatible with observed results. The PCgen algorithm used to obtain GSEM in this study belongs to the recursive causal structure as represented by a directed acyclic graph, DAG, which is a set of variables (nodes) connected by directed edges (arrows) representing direct causal relationships. More precisely, the GSEM can include genetic effects, and it allows for the reconstruction of a causal model attempting to explain the observed data. Notably, these methods do not permit to infer the actual size of causal effects (Buhlmann et al, 2014), while allowing one to rank the importance of variables, and their prioritization with respect to their causal strength (Maathuis et al., 2010). We did not consider population structure in the analysis since statistical inference can become biased under possible model misspecification, such as epistasis (Kruijer, 2016) which has proven to play a role for TKW and GY in durum wheat (Maccaferri et al., 2011). On the other hand, with PCgen, direct genetic effects and structural relations among traits can be inferred, regardless of the population structure and genetic architecture (Kruijer et al., 2020). With GSEM, we revealed that, in our study, the genetic effect on GY was mainly mediated by TKW in non-stress condition, while in case of heat stress GY, NDVI, KWS and HD all showed to be under direct genetic control, then all are expected to be involved in the response to stress and should be taken into account during selection for heat prone environments.

The methods herein employed aimed at identify the variables to be considered in breeding for heat tolerance and therefore reduce the number of secondary traits to be managed either when modeling physiological components of a trait or when selecting those to be considered for genomic prediction (Momen et al., 2018; Arouisse et al., 2021) and breeding. The screening could be based on the target trait or on those genetically correlated with it, as for the indirect selection or for the application of selection indices (Falconer and MacKay, 1996). Recently, the use of selection index in breeding and in genomic selection has been explored in combination with SEM by Hidalgo-Contreras et al. (2021) who concluded that taking into account the causal effects from the structural model notably improved the relative effectiveness of the index with respect to models with no causal information.

The role of environmental covariates is discussed in Arouisse et al. (2021) while Millet et al. (2019) commented on their role in predicting genotypes’ performances in new environments. For this reason, environmental variables are of great interest, besides their use as covariates to correct for spurious associations. In fact, the accurate parametrization of environmental effects is of great importance in “enviromics” (Costa-Neto et al, 2021; Costa-Neto and Fritsche-Neto, 2021; Crossa et al., 2021; Fritsche-Neto et al., 2021) where multi-trait models are integrated with the environmental information into a powerful prediction model (Crossa et al., 2021). The relationship between environmental covariates and the target trait is the first step to pursue the so-called “enviromic” assembly approach (Costa-Neto et al, 2021), which may include different parameters so that genome predictions can be tailored for specific environments. The definition of “envirotyping” by means of a SEM combining both phenotypic and environmental variables provides a powerful framework for synergizing multidisciplinary efforts (Smith et al., 2014) together with other approaches (Porker et al., 2020; Cooper and Messina, 2021), facilitating the discovery of causal pathways as empirical data are tested statistically against the model. Recently, applications of these methods were explored by applying Bayesian estimation for eco-physiological modeling of wheat yield as a function of its component and of weather conditions during key stages of development (Poudel et al., 2022).

This study showed that the response to HS under field conditions of the durum wheat cultivated germplasm worldwide is not homogeneous. Overall, the screening pointed out the presence of a number of genotypes showing relatively tolerant heat stress responses at different developmental and reproductive stages, and more specifically for each of the three main and critical yield-determinants: (i) biomass development/spike number per meter, (ii) spike fertility/kernel number per spike, (iii) grain filling/kernel weight. This finding indicates that breeding strategies aimed at cumulating independent beneficial alleles for different HS tolerance determinants are feasible and could be pursued within the elite germplasm, either by classical breeding or marker-assisted aided breeding. Among the main breeding groups/lineages tested, the best materials in terms of biomass production and fertile culms/spike density, as well as for maintenance of spike fertility were found among the Desert Durum^®^, CIMMYT’80 and ICARDA breeding pools. This observation confirms recent finding by Emebiri et al. (2023) who identified several heat-stress tolerant CIMMYT’80 genotypes in a controlled environment screening of durum germplasm specifically targeted for heat-induced floret sterility due to controlled heat exposure at heading/flowering. This finding is overall relevant for future breeding, in view of the expected impact of increased average temperatures due to Global Climate Change on durum wheat production areas such as the Mediterranean Basin. In this respect, it is appropriate to point out that in practical breeding improving a single specific component of heat stress tolerance is not resolutive and could even lead to detrimental side effects. In this case, maintenance of fertility under stress should necessarily be associated to maintenance of physiological senescence and translocation processes and hormonal balance associated to a regular, not accelerated grain filling (Ashfaq et al., 2022; Xu et al., 2022).

This study allowed us to analyze the response to heat stress of a panel of durum wheat genotypes representing a relevant portion of the genetic diversity available worldwide. We identified in the panel a large variability for the performance under heat stress, which will provide a valuable source of alleles/haplotypes for adaptation to heat-prone environments. We also considered the relations among plant variables and environmental covariates identified by means of a SEM, confirming that heat stress affects yield mostly after anthesis. The study of structural models, and in particular of GSEM, lays a foundation for the next steps in elucidating the physiological response to heat by mapping QTLs controlling the trait (Igolkina et al., 2020) and to implement either marker-assisted selection, if large effects QTLs are identified, or genomic prediction tailored to heat tolerance improvement of durum wheat (Costa-Neto et al., 2021). Most importantly, we believe that meaningful tools to link genetic knowledge and environmental specificity, such as those employed in the present study, can be part of the most needed translational research aimed at actually improving crop cultivars (Kole et al., 2015; Millet et al., 2019; Reynolds et al., 2021; Welcker et al., 2022; Della Coletta et al., 2023; Sall et al., 2024).

In conclusion, the dissection of traits involved in response to heat showed that the effects of HS were particularly pronounced for NDVI, KWS, GFP, and GY. Combination of plant and weather related variables in GSEM modeling suggested that the causal model of performance under HS directly involves genetic effects on GY, NDVI, KNS and HD. Among factors determining response to heat stress, environmental variables have a role and could be integrated in multi-trait prediction models. We identified consistently suitable sources of genetic resistance to heat stress to be used in different durum wheat pre-breeding programs. Among those, Desert Durums and CIMMYT’80 germplasm showed the highest degree of adaptation and capacity to yield under high temperatures and can be considered as a valuable source of alleles for adaptation to breed new HS resilient cultivars.

Finally, the next steps should include mapping genomic regions and characterizing QTLs controlling the traits involved in performance under high heat stress and/or developing multi-trait selection criteria to predict genotypes that could be best adapted to heat-prone environments. From a breeding standpoint, and given the wide genetic diversity of the present panel, and the fact that genotypes with low reduction in GY and related traits were identified in all of the germplasm groups herein tested, the identification and possibly cloning of heat tolerance QTLs from the most representative collections of durum germplasm publicly available (Maccaferri et al., 2019; Mazzucotelli et al., 2020) will eventually allow breeders to systematically accumulate the favorable alleles/hapotypes with outstanding performance under heat conditions.

## Data availability statement

The original contributions presented in the study are included in the article/**Supplementary Material**, further inquiries can be directed to the corresponding author.

## Author contributions

EG: Conceptualization, Writing – review & editing. EF: Conceptualization, Writing – review & editing. MM: Conceptualization, Writing – review & editing. KA: Conceptualization, Writing – review & editing. RT: Conceptualization, Writing – review & editing.

## Glossary

**Table:**

CENEB	Campo Experimental Normal E Borlaug
CIMMYT	Centro Internacional de Meyoramento de Maize Y Trigo
DTM	Date To Maturity
GFP	Grain Filling Period
HD	Heading Date
HS	heat-stress trial, delayed sowing
LASSO	last absolute Shrinkage and selection operator
ME	Mega Environment
MTM	Multiple Trait Model
NS	non-stress trial
QTL	Quantitative Trait Locus
RIL	Recombinant Inbred Line
SEM	Structural Equation Model
GSEM	Genetic-SEM
SOM	Soil Organic Matter
UNIBO	University of Bologna
***Environmental parameters***	
AMT	Average Maximum Temperature
GDD	Growing Degree Days
HDD	Heat Degree Days
HDD_A	HDD during Anthesis
HDD_GF	HDD during Grain Filling
NDTH30	Number of Days with Temperature > 30°
NDTH35	Number of Days with Temperature > 35°
***Phenotypic traits***	
GY	Grain Yield
IT_INDEX	NDVI area under heat stress progress curve
KLE	Kernel Length
KNS	Kernel Number per Spike
KWI	Kernel Width
KWS	Kernel Weight per Spike
NDVI	Normalized Difference Vegetation Index
PH	Plant Height
SKT	number of spikelets per spike
SPM	number of Spikes per linear meter
TKW	Thousand Kernel Weight
TWT	Test Weight
