# Supplementary material for: Dissecting the effect of heat stress on durum wheat under field conditions
Source: Front Plant Sci. 2024 Jun 28;15:1393349. doi: 10.3389/fpls.2024.1393349 (PMC11239346; doi:10.3389/fpls.2024.1393349)
Supplement: Supplementary file 1 [file Image_1.pdf]

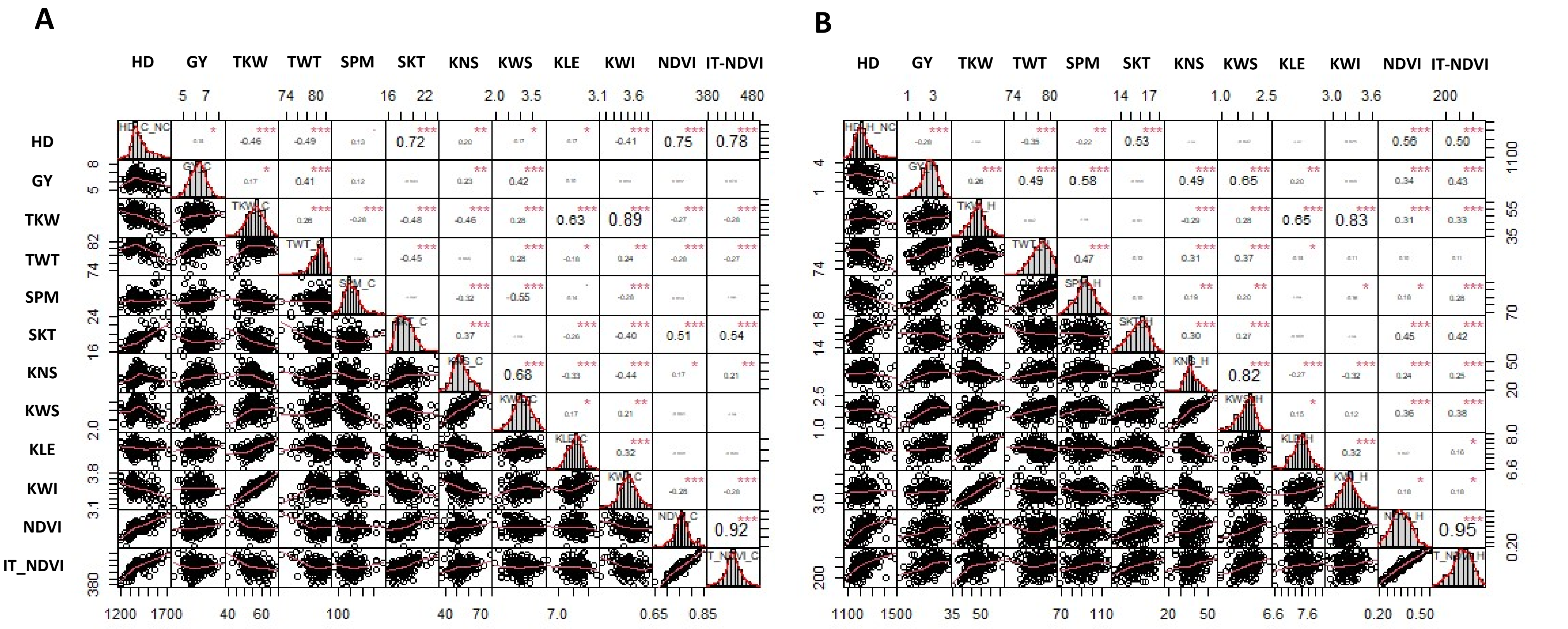

Supplementary Figure 1. Pearson correlation coefficient between traits observed in a durum diversity panel (N=187) evaluated at CENEB-Cd Obregon, Mexico in 2018 and 2019 (values are 2-years average BLUEs). Traits: days to heading date (HD), grain yield (GY), thousand kernel weight (TKW), test weight (TWT), spikes per linear meter (SPM), spikelets per spike (SKT), kernel number per spike (KNS), kernel weight per spike (KWS), kernel length (KLE), kernel width (KWI), normalized difference vegetation index (NDVI), and area under heat stress progress curve for NDVI (IT_NDVI) . **(A)** Early sowed Non-Stressed control condition. **(B)** Late sowed Heat Stress condition.
