# Supplementary material for: Dissecting the effect of heat stress on durum wheat under field conditions
Source: Front Plant Sci. 2024 Jun 28;15:1393349. doi: 10.3389/fpls.2024.1393349 (PMC11239346; doi:10.3389/fpls.2024.1393349)
Supplement: Supplementary file 3 [file Image_3.pdf]

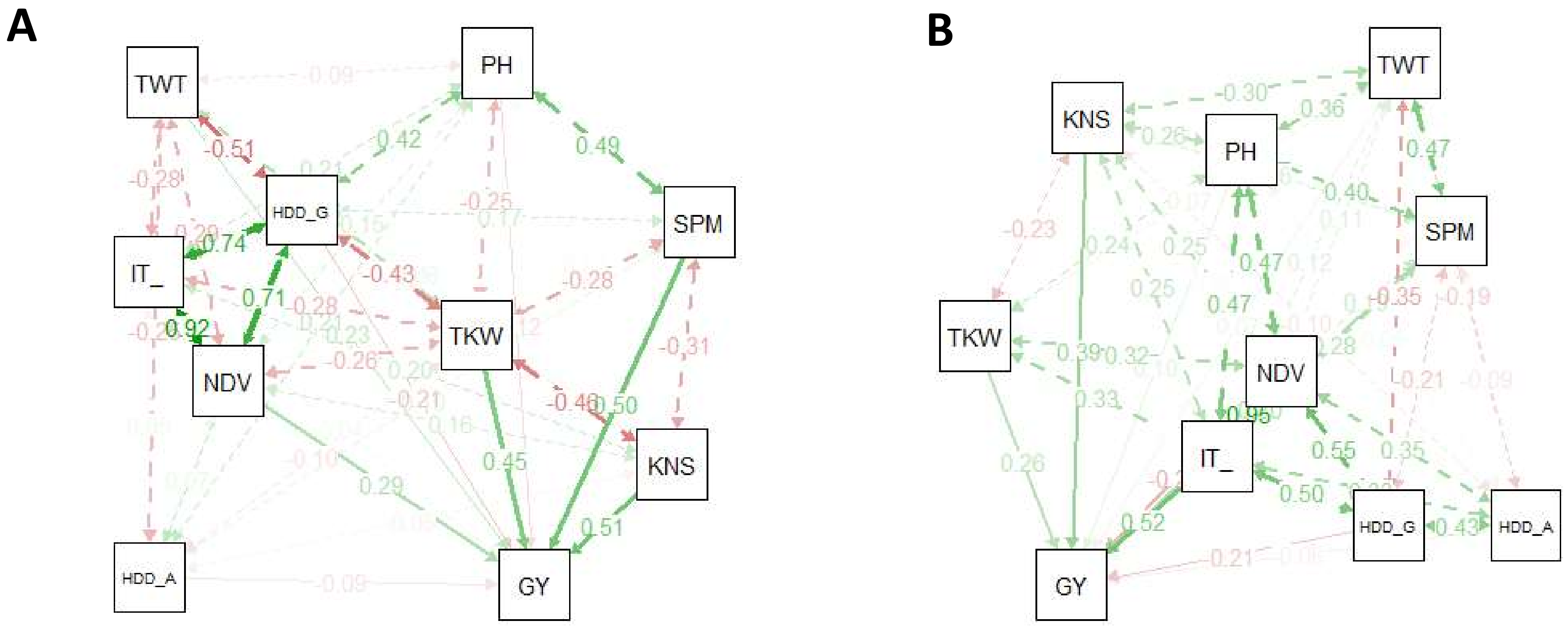

Supplementary Figure 3. Structural equation models (SEM) from yield trials involving the UNIBO-Durum Diversity Panel evaluated at CENEB-Cd. Obregon, Mexico, in 2018 and 2019. Traits considered were grain yield (GY), as the dependent variable, plant height (PH), thousand kernel weight (TKW), test weight (TWT), spikes per linear meter (SPM), kernel number per spike (KNS), normalized difference vegetation index (NDVI), heat degree days at anthesis (HDD_A) and heat degree days at grain filling period (HDD_GF). Significant (α=0.01) functional relationships in the SEM are represented as single-headed arrows, whereas correlative relationships are represented with double-headed arrows. Standardized path coefficients are shown and also represented by arrows, with color denoting direction similar to the correlation matrix, and the thickness indicating relative coefficient size, with thicker arrows denoting larger coefficients. **(A)** Early sowed Non-Stressed control condition. **(B)** Late sowed Heat Stress condition.
